# Building capacity for dissemination and implementation research: one university’s experience

**DOI:** 10.1186/s13012-017-0634-4

**Published:** 2017-08-16

**Authors:** Ross C. Brownson, Enola K. Proctor, Douglas A. Luke, Ana A. Baumann, Mackenzie Staub, Matthew T. Brown, Mallory Johnson

**Affiliations:** 1 Prevention Research Center in St. Louis, George Warren Brown School of Social Work, Washington University in St. Louis, St. Louis, MO 63130 USA; 2 Division of Public Health Sciences and Alvin J. Siteman Cancer Center, Department of Surgery, Washington University School of Medicine, Washington University in St. Louis, St. Louis, MO 63110 USA; 3 Center for Mental Health Services Research, George Warren Brown School of Social Work, Washington University in St. Louis, St. Louis, MO 63130 USA; 4 Institute for Public Health, Washington University in St. Louis, St. Louis, MO 63130 USA; 5 Center for Public Health Systems Sciences, George Warren Brown School of Social Work, Washington University in St. Louis, St. Louis, MO 63130 USA; 6 Brown School Evaluation Center, Washington University in St. Louis, St. Louis, MO 63130 USA

**Keywords:** Capacity building, Capability development, Dissemination and implementation research, Knowledge transfer, Organizational capabilities, Translational research

## Abstract

**Background:**

While dissemination and implementation (D&I) science has grown rapidly, there is an ongoing need to understand how to build and sustain capacity in individuals and institutions conducting research. There are three inter-related domains for capacity building: people, settings, and activities. Since 2008, Washington University in St. Louis has dedicated significant attention and resources toward building D&I research capacity. This paper describes our process, challenges, and lessons with the goal of informing others who may have similar aims at their own institution.

**Activities:**

An informal collaborative, the Washington University Network for Dissemination and Implementation Research (WUNDIR), began with a small group and now has 49 regular members. Attendees represent a wide variety of settings and content areas and meet every 6 weeks for half-day sessions. A logic model organizes WUNDIR inputs, activities, and outcomes. A mixed-methods evaluation showed that the network has led to new professional connections and enhanced skills (e.g., grant and publication development). As one of four, ongoing, formal programs, the Dissemination and Implementation Research Core (DIRC) was our first major component of D&I infrastructure. DIRC’s mission is to accelerate the public health impact of clinical and health services research by increasing the engagement of investigators in later stages of translational research. The aims of DIRC are to advance D&I science and to develop and equip researchers with tools for D&I research. As a second formal component, the Washington University Institute for Public Health has provided significant support for D&I research through pilot projects and a small grants program. In a third set of formal programs, two R25 training grants (one in mental health and one in cancer) support post-doctoral scholars for intensive training and mentoring in D&I science. Finally, our team coordinates closely with D&I functions within research centers across the university. We share a series of challenges and potential solutions.

**Conclusion:**

Our experience in developing D&I research at Washington University in St. Louis shows how significant capacity can be built in a relatively short period of time. Many of our ideas and ingredients for success can be replicated, tailored, and improved upon by others.

**Electronic supplementary material:**

The online version of this article (doi:10.1186/s13012-017-0634-4) contains supplementary material, which is available to authorized users.

## Background

Dissemination and implementation (D&I) science has grown at a rapid pace over the past 15 years in the USA and several other countries. Grounded in part by an early paper by Lomas [1], a milestone in the development of D&I science was the issuance of the first program announcement on Dissemination and Implementation Research in 2002 [2]. Another key activity was development of the Veterans Administration’s Quality Enhancement Research Initiative (QUERI), launched in 1998, as part of an effort to more quickly and consistently translate research knowledge into practice to improve healthcare for Veterans [3, 4]. In 2006, the *Implementation Science* journal was founded, providing the first scientific venue focused solely on D&I research.

As these initiatives have developed, it became apparent that for D&I science to thrive, greater capacity is needed in individuals and institutions conducting research [5–8]. Capacity building is a process occurring in individuals, organizations, and systems that results in higher levels of skills and abilities to carry out and disseminate high-quality research [9–13]. Similar to how ecological levels influence health behaviors [14], multiple levels (from individuals to systems) influence capacity and these influences interact across levels.

Since 2008, Washington University in St. Louis has dedicated significant attention and resources toward building D&I research capacity. This paper describes our process, challenges, and lessons with the goal of informing others whom may have similar aims at their own institution. This story highlights the importance of connecting people with different disciplinary traditions who are involved in D&I research in various capacities and settings, and encouraging a wide variety of research capacity building activities.

## Building blocks for enhancing D&I research capacity

For any new field to prosper, both human and intellectual capital must be developed to discover knowledge and narrow the research to practice gap. This section briefly highlights how D&I research capacity can be developed across three inter-related domains (people, settings, and activities).

### People: mentoring and interdisciplinary collaboration

Mentoring has been shown to have clear and numerous benefits (in particular research productivity, career satisfaction, and career success [15, 16]). This is true for the individual being mentored [17] as well as the mentor [18–20]. In several national-level training programs in the USA (e.g., the Implementation Research Institute (IRI) [21], Mentored Training in Dissemination and Implementation Research for Cancer (MT-DIRC) [22]), the connection between mentors and trainees is central to development of scholars [23]. In these programs (described later in this article), a mentor works with a trainee over a 2-year period.

Dissemination and implementation science has no single disciplinary home, drawing on multiple fields. The benefits to D&I research from crossing disciplines are many, including the need for D&I theories to be interdisciplinary, the likelihood that D&I measures and methods will stem from multiple disciplines, and the value in building practice collaborations for D&I research that cross sectors (e.g., public health, social services) [24]. Interdisciplinary approaches, such as team science, help to bridge disciplines and break down ‘silos’ often caused by funding mechanisms and organizational structures (e.g., a cancer epidemiologist may not naturally interact with a social science researcher in mental health) [25–27].

### Settings: organizational commitment

Organizational climate and culture are fundamental issues in developing and conducting a D&I study [28, 29]. Within organizations conducting D&I research (often academic institutions), several key ingredients foster the building of organizational capacity. Since the field of D&I research often involves projects with long time horizons and numerous disciplines, researchers, especially junior investigators, need to be afforded adequate support and time to show progress. Academic leaders at all levels need to recognize that D&I research is fundamental to the missions of universities (i.e., showing an impact in society) and therefore devote resources commensurate with this charge. When these characteristics are present, organizations support collective capacity through mutual support, information sharing, and collaboration [30].

### Activities: trainings, tools, and toolkits

As the field of D&I research advances, increasing opportunities for training becomes essential to meeting the full potential of the discipline to improve population health in an efficient and timely manner. Over the past decade, training opportunities for D&I research have emerged, including immersive training institutes, 1-day workshops, academic graduate programs, individual academic courses, webinar series, and career development awards [5].

Tools and toolkits assist D&I researchers in numerous ways. Most often they are skill-based and focus on how to (1) identify and use theories, frameworks, and models [31, 32]; (2) select reliable and valid measures [33, 34]; (3) identify and apply D&I strategies [33, 34]; (4) select appropriate evaluation approaches [35, 36]; and (5) disseminate study findings to various audiences [35, 36]. Other toolkits focus on more general competencies such as grant writing, the grant review process, and getting published [37–39].

## Building D&I capacity in a university setting

**Fig. 1:**
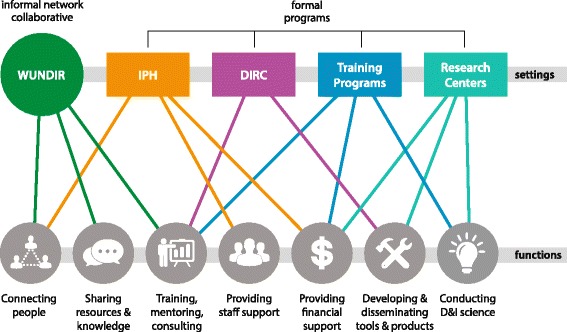
Conceptual framework demonstrating interconnected settings and functions that supported development of dissemination and implementation science research capacity at Washington University in St. Louis

The development of D&I science at Washington University in St. Louis has drawn upon the skills, commitment, and leadership from many individuals along with funding from university and federal partners. It covers all three domains (people, settings, activities) noted above. As Fig. 1 illustrates, a set of formal and informal institutional settings were built and used to support a wide variety of D&I related administrative, training, and scientific activities. The breadth of activities and the overlapping institutional supports were critical to the success of the Washington University D&I initiative.

### Washington University Network for Dissemination and Implementation Research

The Washington University Network for Dissemination and Implementation Research (WUNDIR) was created in 2010 to provide individuals interested in D&I science with an informal research network collaborative. The group was conceived and launched after an initial meeting of three senior faculty (EKP, RCB, DAL) who recognized the value of regular networking to build on their distinct but complementary foci in D&I science. WUNDIR started as a small group of people who reviewed abstracts for the NIH D&I conference. While participation grew quickly, the group has maintained a grass roots culture, with meeting content shaped in response to member needs and interests. In particular, WUNDIR was designed from the outset to provide a more informal setting for D&I discussion and collaboration, in contrast to the more formal institutional settings described below. Senior faculty rotate responsibility for hosting, nominating topics and presenters for meeting agendas, and paying for modest refreshments. This pattern persists, although the Washington University Institute for Public Health began to provide a “home” for WUNDIR in 2014. Since WUNDIR’s inception and as of March 2017, a total of 284 individuals have attended at least one WUNDIR meeting. Of those individuals, 214 are affiliated with Washington University, 56 are from external institutions, and 14 have missing information. WUNDIR participants have included D&I interested scholars in all phases of their careers (e.g., doctoral students, post-doctoral fellows, research staff, junior, and senior faculty), representing multiple schools and departments across campus, including social work, public health, medicine, pharmacy, and psychology.

WUNDIR meetings help to introduce D&I concepts to those new to the field and forge a transdisciplinary understanding of the methodological issues and conceptual challenges required for D&I scholarship. Typical agendas include: *welcome and introductions* including member report of publication and grant updates (most meetings have a few first-time participants), *news and updates* (updates on the field at large, upcoming conferences and training opportunities, new funding opportunities etc), a *D&I methods presentation* and critique, discussion of *works in progress*, and three levels of *grant review* (big picture, early drafts, and mock peer review). The agendas are built by soliciting the membership for agenda items; typically individuals nominate items they will present for themselves, general membership provide input via simple surveys and facilitated brainstorming, and senior investigators nominate items and presenters based on their knowledge of the needs and abilities of the members. Members represent a wide variety of settings and content areas (e.g., mental health services, public health, acute care, emergency medicine, cancer, tobacco, and HIV). As of March 2017, WUNDIR had 49 members (membership is defined as having attended at least 2 meetings including at least 1 meeting in the past year) and the group meets every 6 weeks for half-day sessions (attendance ranges from 20 to 40 people per meeting). Meeting sites rotate between the university’s two campuses (medical school and Danforth campus, separated by 2 miles and a large urban park) and across days of the week, so as to avoid recurring conflicts with members’ teaching and clinical schedules.

**Fig. 2:**
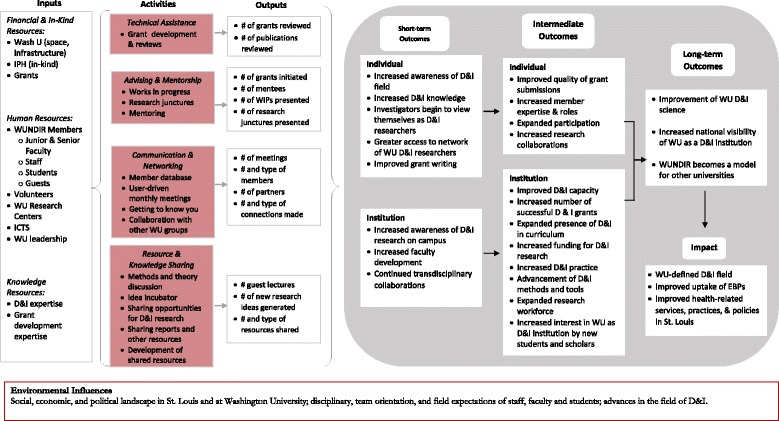
Washington University Network of Dissemination and Implementation Researchers (WUNDIR) logic model

In February 2016, WUNDIR leadership developed a WUNDIR logic model as part of a systematic process evaluation (Fig. 2). The model provides a visual roadmap showing how inputs (e.g., resources and knowledge) and activities of WUNDIR link to the outputs (or products) and anticipated outcomes. Outcomes are expected at both the individual (e.g., improved grant writing) and organizational (e.g., expanded D&I research workforce) levels. Based on this logic model, and utilizing both quantitative and qualitative evaluation data, the evaluation sought to answer these questions: (1) what has been the reach of WUNDIR?; (2) to what extent do members find WUNDIR useful?; (3) how has WUNDIR helped to strengthen members’ D&I research?; and (4) what new directions can WUNDIR take?

An online survey collected data from WUNDIR members (*n* = 145) on D&I network strengths, areas of improvement, and suggestions for the future. A total of 40 surveys were completed in their entirety, with three additional partial completions (response rate = 29.7%). Forty-eight percent of participants attended four or more WUNDIR meetings in 2015, and 40% of survey participants had been involved in WUNDIR for over 3 years. In addition to the quantitative survey, the evaluation team conducted 13 in-depth interviews with regular (*n* = 8) and irregular (*n* = 5) WUNDIR members. Participants in both the survey and interviews included students, staff, and faculty. The online survey and qualitative interview guide are available as Additional files 1 and 2.

Evaluation participants reported that participation in WUNDIR increased their knowledge of D&I science (within and outside the university), led to new professional connections and enhanced skills such as grant and publication development. The most useful components of WUNDIR included opportunities for building new collaborations, conducting informal networking, participating in grant reviews, obtaining mentoring, and discussing works in progress.

Qualitative data from interviews provided additional richness to many of the survey findings.

Active members felt that exposure to new topics and the opportunity for hands-on learning was an important benefit of membership. For example,

You’re exposed to the latest and most relevant literature. At every WUNDIR meeting there is an article on methods or a data analysis procedure we’re all learning from.

If I were to write a D&I grant I know a lot more about where to find the frameworks, the importance of using a framework, both for the planning of the study and for the measurement.

WUNDIR has created a welcoming and open environment for researchers of all levels.

It’s very inclusive. Everybody gets a change to talk and hear opinions.

I think that just being part of this community within our larger Washington University community is a really nice thing. It feels good, it feels like you’re supported, and challenged, and not doing this work in isolation.

WUNDIR builds technical skills and strengthens D&I projects through hands-on reviews. Activities like the mock grant reviews provide members with an opportunity for feedback, ultimately strengthening their final products.

It [mock reviews] advances people’s knowledge, it offers opportunity for feedback and review, and it strengthens everyone’s work. I think it gives people a place to start if they are interested in D&I work.

It very clearly strengthens capacity in terms of strengthening the quality and

also the volume of D&I grants or D&I related grants that go out. Parallel to that,

not only do proposal reviews get supported, but also papers in development

and other projects.

The D&I work facilitated by WUNDIR is recognized by researchers and trainees around the country.

At the very least [WUNDIR] is one of the first of its kind. I think that has really caught the interest and admiration of others outside of the university and others have started to model similar types of networks within their institutions. I think that providing that model has really been critical to the field of D&I and others taking similar approaches.

The survey and interviews also identified several new directions for WUNDIR that could enhance the vibrancy, reach, and impact of WUNDIR. Recommendations included a need to create a stronger online presence, increase marketing and outreach efforts, connect with D&I practitioners and implementers, create alternative D&I engagement opportunities, increase student engagement, and reach out to more schools and departments at Washington University.

The success of WUNDIR has spawned two other related spin-off groups at Washington University: the Network Science Interest Group and the Collaborative of Health Economics and Policy Analysis Researchers. Both multidisciplinary groups are building on the lessons learned from WUNDIR and also contributing to D&I science. Other related interest groups and discussion forums in D&I research are held at numerous other institutions including the University of North Carolina-Chapel Hill, University of Washington, San Diego State University, University of Colorado, and University of Pennsylvania.

### Dissemination and Implementation Research Core

The Dissemination and Implementation Research Core (DIRC) began in 2007 as a pilot program within the Washington University Institute for Clinical and Translational Science (ICTS), and was our first formal component of D&I infrastructure. The faculty team proposing the core argued the importance of D&I science to ICTS goals, and framed the core as a technical assistance resource similar to other cores focused on earlier stage translation. Since its inception, the DIRC has been supported through 2 cycles of ICTS funding. The budget is modest but the core’s establishment provides visibility, a core of funding for key activities, and a variety of supports (e.g., communication, website) common to all ICTS cores.

The mission of DIRC is to accelerate the public health impact of clinical and health services research at Washington University by increasing the engagement of ICTS investigators in later stages of translational research. DIRC aims to (1) advance D&I science within the ICTS; and (2) develop and equip ICTS researchers with tools for D&I research. DIRC is led by a director (EKP), a team of core faculty (RCB, DAL, Graham Colditz), and a PhD-level coordinator (AB). The DIRC is further supported by a team of research assistants specializing in D&I science, typically including 2 PhD students and 1–2 masters students. The core faculty is responsible for the scientific direction of the core, resource procurement and allocation, substantive and methodological guidance, quality control of products, and dissemination of scientific information (presentations, brown bag seminars, articles). The coordinator is responsible for triaging and supporting customers, arranging peer review of proposal drafts, tracking services, and supervision of research assistants. The research assistants structure and conduct searches for literature, measures, and design options in response to investigator needs; contribute to peer review of grant proposals; and update the core’s collections.

To accomplish aim 1 (advance the science), DIRC conducts two main activities: presentations/meetings and consultations with DIRC customers. Presentations seek to increase the visibility and understanding of D&I research. The DIRC director and members of the core faculty make numerous presentations about D&I science in such places as ICTS meetings, with university senior officials, departmental grand rounds, classes, and seminars for ICTS fellows and various training program, both within Washington University and with ICTS participating organizations. In addition, DIRC faculty members promote collaboration among the growing base of ICTS investigators through WUNDIR.

Consultation is the central activity for DIRC core faculty members (see common questions and related challenges in Table 1). Most often, this begins with review of potential research aims of the grants that typically leads to one of two different scenarios. First, if early consultation suggest the project is testing efficacy or effectiveness, then the principal investigator is informed that D&I research questions may be premature. In these cases, investigators are referred to other ICTS cores and informed that “pre-implementation” work may be appropriate in setting up a future D&I study. Second, if consultation suggests a potential D&I study, DIRC provides several services to advance the grant application. These include helping the investigator refine aims and providing conceptual and methodological consultation.

**Table 1:** Questions posed by individuals providing advice and challenges that are common in DIRC consultations

Domain	Key questions	Potential challenges
1. Main interest/research question(s)	What are you seeking to accomplish? Test a prevention strategy or treatment Implement an evidence-based intervention Disseminate a new intervention/treatment	Too many questions for one study Not a D&I study
2. Background	Have you conducted a study prior on the program/policy/treatment/intervention? Efficacy Effectiveness Service system Organizational context Treatment/intervention adaptation Early phase D&I study Scale-up potential training	Little efficacy/effectiveness data on intervention
2. Evidence-based intervention to be implemented	Is the evidence for the program, treatment, or set of services to be implemented demonstrated?	The intervention may not have been proved/tested
3. Care, burden, or quality gap	What is the quality gap in your program of research/in the study that you are proposing?	The quality gap has not been well documented The prevented fraction has not been estimated
4. Setting	1. Who are consumers/patients/clients/stakeholders? 2. Who are the key stakeholders in the implementation and how are they engaged in the proposed study? 3. Who are the providers? What is their level of exposure, training to the intervention of interest? What are the training possibilities? Training challenges? 4. How universal or generalizable is the setting of delivery? (e.g., part of a national system, or network?)	Lack of data on organizational level providers Multilevel interventions many involve many different providers
5. Study design	1. How would you describe the study design? Observation of a naturally occurring implementation/dissemination plan to introduce (manipulate) something new (using an implementation or dissemination strategy, comparing the effectiveness of two implementation strategies) 2. What methods will you use? Quantitative only Qualitative only Mixed methods	No local expertise on qualitative methods
6. Conceptual model and theoretical justification	Do you have a clear conceptual framework/theory/model that informs the design and variables being tested? Does your conceptual model frame your evaluation?	Little to no knowledge about conceptual models
7. Outcomes	What outcomes are you thinking about evaluating?	Reliable and valid methods for measuring outcomes do not exist
8. Strategies	What are the strategies you are thinking about using to implement the intervention?	Lack of data on effective strategies

Aim 2 (provide D&I tools) is closely related to Aim 1 and provides ICTS-affiliated researchers with the necessary research tools and resources to understand basic principles, theories, and common tools of D&I science. The specific tools have evolved over time, starting with literature reviews and lists of measures for implementation constructs, often provided within the context of one-to-one consultation with investigators. To increase the efficiency of the core, tools have become more formalized within the past year. A series of toolkits which are designed for independent use have been developed by DIRC investigators. To date, toolkits have been developed across eight content areas ranging from introductory to advanced topics in D&I research (Table 2). These toolkits have been particularly useful in coaching investigators prior to and between in-person consultation visits.

**Table 2:** Toolkits for D&I research developed by DIRC

Topic	Description
Introduction to D&I science	Introductory material that includes an overview of the field and terminology
Aims	Guidance on how to write effective aims
Barriers and facilitators	Information on how to identify and measure barriers and facilitators
Research designs	Brief overview of research designs (experimental, quasi-experimental) for D&I studies
Strategies	Commonly used D&I strategies, including recommendations for reporting in manuscripts
Organizational measures	Information on organizational constructs and measures to be addressed in a D&I study
Outcomes	Guidance on which D&I outcomes to include in a study
Key ingredients in grant proposals	Adapted from Proctor et al. [38], provides 10 ingredients for a successful grant application

Website link: https://sites.wustl.edu/wudandi/di-toolkits/

Numerous outcomes are directly or indirectly linked with DIRC activities. The number of consultations provided to CTSA investigators is one important metric. The number of investigators served has grown steadily, from 11 in 2009 to around 30 per year since. These investigators have submitted 98 grants, with DIRC support, including small pilots, career awards, NIH, AHRQ, and PCORI grants. Of these, 46% (*n* = 47) have been funded. Other outcomes include new tools and courses in D&I science. Two new courses have been developed, a one credit *Introduction to D&I science* and a three credit *Implementing and evaluating evidence-based practices*. In addition to numerous papers, three members of the DIRC core faculty edited the book *Dissemination and Implementation Research in Health: Translating Science to Practice* [40], with many Washington University investigators as authors. One of the first D&I cores within Clinical and Translational Science Awards across the country, the DIRC has also strengthened the visibility of Washington University’s ICTS and helped position the university as a national leader in the D&I field.

### Center for Dissemination and Implementation, Institute for Public Health

In 2011, The Washington University Institute for Public Health established a Center for Dissemination and Implementation to support the D&I of health interventions. The Center Director (EKP), a full-time Center Manager (MB), and a master’s research fellow (MJ) carry out the work of the center.

The Center oversees and support two funding programs: a pilot projects program (1 year, $30,000 direct costs) and a small grants program (1 year, $7500 direct costs). Supported by a 3-year grant from the Chancellor’s office of the University, the grants programs were established in response to needs identified in an early WUNDIR needs assessment. These programs enable investigators to acquire preliminary data for subsequent grant proposals submitted to external funders.

The center also supports a campus-wide seminar series which has increased the visibility of D&I science at Washington University and its affiliated hospitals. Perhaps even more importantly, in conjunction with seminar series speakers—each a leading national or international expert in D&I—the center manager arranges consultation sessions between the speaker and researchers working actively on grant submissions.

Comments on seminar series and consultations:

I met with [consultant] after sending a draft application for the CTSA Innovation Award. He had reviewed it prior to our meeting and provided very valuable insights and suggestions”… (specifically related to …innovative research methods) “as well as recommending a possible collaborator. The proposal received excellent reviews at study section, in part due to his input.

The consultation allowed hospital system partners directly involved with integrating qualitative data with quantitative data the opportunity to learn more about relevant frameworks for implementation and evaluation of different methods for integration.

An annual Next Steps in Public Health: D&I Proposal Development Bootcamp is designed to stimulate development of innovative grant proposals in implementation science and prepare them to compete successfully for external funding. The *bootcamp* is a single day of intense consultations tailored to each team. The bootcamp matches research teams with up to 9 separate consultation sessions with national and local experts on any of over 50 topics relevant to D&I grant writing. In the first 2 years, 32 investigator teams represented 19 Washington University divisions or departments of 3 schools, 14 external organizations, and 2 health care settings/hospitals submitted applications for this event. Teams included 17 investigators new to the center network.

In 2017, the center launched a Training in Implementation Practice Leadership (TRIPLE) program for clinical leaders in local community agencies. The Center Director, a public health faculty member, and a national expert led the training, organized by the center manager. The three half-day sessions focused on how to advance the adoption and sustainability of evidence-based interventions agencies wished to deliver. Sixteen trainees from 8 local agencies participated in the training in its inaugural year.

Finally, the center supports WUNDIR by soliciting agenda items for the regular meetings, identifying and soliciting reviewers for grant review requests, orchestrating the rotation of meeting refreshments, tracking attendance, and building the WUNDIR email list.

### National/international training programs

Through two R25 training grants (one in mental health and one in cancer) supported by national funders, post-doctoral scholars across the United States of America and other countries are selected for intensive training and mentoring in D&I science. Across these two training programs, a today of 83 fellows have been trained (as of March 2017).

#### The Implementation Research Institute (IRI)

The IRI is a 2-year training institute in mental health implementation science, supported by the National Institute of Mental Health, the Department of Veterans Affairs, and the National Institute on Drug Abuse [21, 23, 41]. Now in its second round of (5-year) funding, the IRI has trained 43fellows. Drawn from a national pool of applicants, fellows attend two annual weeklong trainings at Washington University in St. Louis, travel for a site visit on still-in-the-field implementation projects, and attend implementation science conferences.

#### Mentored Training for Dissemination and Implementation Research in Cancer (MT-DIRC)

Similar to IRI, MT-DIRC is also a 2-year training program supported by the National Cancer Institute and the Department of Veterans Affairs [21]. In MT-DIRC, 14 fellows per year attend two annual weeklong trainings at Washington University in St. Louis and are linked with a senior scholar in D&I science for mentoring over a 2-year period. To support these efforts, the core team developed and refined a set of competencies [42, 43] and model curriculum in D&I research and is actively disseminating program components for adoption by other individuals and institutions.

### D&I functions within research centers

Our team has collaborated closely with other Washington University investigators as they develop center grants, research cores, or other entities where D&I science is critical to their objectives. Many of these activities provide foundational supports that endure beyond any single project or grant. Federally supported centers with significant D&I foci have included a Center for Mental Health Services Research (principal investigator: E. Proctor), Center for Diabetes Translation Research (principal investigator: D. Haire-Joshu), the Transdisciplinary Research on Energetics and Cancer grant (principal investigator: G. Colditz), the Health Communication Research Laboratory (principal investigator: M. Kreuter), the Center for Public Health System Sciences (principal investigator: D. Luke), and the Prevention Research Center in St. Louis (principal investigator: R. Brownson).

## Making it happen: the academic trade-offs

Challenges encountered in building D&I research capacity can occur at multiple levels and time points, but our collective experience over the past decade, results from our internal evaluations, and the existing literature provide a number of lessons and strategies for addressing these challenges. While not exhaustive, this inventory of key issues and lessons should aid others who seek to replicate all or part of our approach.

### Challenge 1: lack of awareness about D&I and how it is defined

In providing initial D&I consultation, perhaps the greatest challenge is helping investigators understand the complex, dynamic nature of D&I science. Common issues encountered include the proposed project not being a D&I study or too many questions or aims for one study (Table 1).

Strategies and lessons

- Lay the groundwork for D&I science. We spent considerable time in the early years of this process presenting foundational informational sessions to various audiences across campus. This was a challenge in that this time was often not directly “paid for,” but it was essential in building awareness and education among future partners on the value of D&I science for their work.
- Utilize efficient decision aids. To initially determine whether a proposed project is a D&I study, we have developed a checklist and decision tree. We also provide key implementation articles to individuals seeking advice, including those identifying essential components of implementation research grant proposals [38], conceptual frameworks [44, 45], and articles that help investigators anticipate the potential D&I outcomes for evaluation in the proposal [46].

### Challenge 2: the broad scope of D&I science

Working across a research-intensive university, we encounter a wide breadth of scientific interests and experience among investigators. We consult with investigators ranging from post-docs to endowed professors and department chairs. Their substantive foci span pediatrics, public health, social services, cancer screening, rehabilitation, metabolic health, emergency medicine, intensive care, and transplantation.

Strategies and lessons

- Promote active participation across disciplines. Interdisciplinarity is a core element of D&I science [44]. However, at Washington University, taking an interdisciplinary approach to building D&I capacity had more practical effects. By starting out as an interdisciplinary collaborative, WUNDIR was able to be a home for any scientist no matter where they lived in the university. This was communicated in a number of ways—by the variety of meeting topics, by rotating meetings between the various campuses, by explicitly recognizing how other disciplines could enhance research ideas, and by featuring speakers representing various theoretical and methodological traditions. Essentially, the early interdisciplinary focus allowed us to endow WUNDIR with a culture of inclusiveness, which we feel was crucial to its acceptance and eventual success. A cross-disciplinary approach is also effective in reducing the need for every team member to be an expert in D&I science. For example, by involving our DIRC methods core, not every principal investigator needs to become a D&I scholar. Core faculty and staff must listen carefully, learn, identify the core D&I scientific challenge, and draw on a wide range of literature and research methods.

### Challenge 3: the need to maintain resources

Even in a university like ours where support for D&I research is strong, an ongoing challenge in maintaining capacity has been the need to support D&I activities with relatively small funding sources and varying budget periods, making it difficult at times to set long-term goals and plans.

Strategies and lessons

- Garner institutional commitment and share ownership. At Washington University, we have had strong institutional support for D&I science across many levels including the ICTS, the Institute for Public Health, the Brown School of Social Work, the Siteman Cancer Center, and the Chancellor’s office. University leaders have been enthusiastic and generous, enabling us to support a full-time staff member, a part-time staff member, and numerous part-time research assistants. While the terminology of D&I research can be cumbersome [47], the ultimate goals of D&I (e.g., showing impact in society, connecting research to practice and policy, informing teaching) are fundamental to every academic institution. The support across our institution has also allowed us to share ownership of D&I research across multiple schools and departments.

### Challenge 4: the need for academic leadership and networking

Developing D&I research capacity requires consistent involvement of mid- to senior-level faculty members. Given that D&I science is a relatively new field [48], most universities will encounter a deficit in academic leaders who can take on all of the issues outlined in this article. Moreover, as new grants are awarded, the effort levels of mid- and junior-level investigators increase, making faculty leaders at many levels busier and less available. When local D&I leadership is lacking, creative approaches to networking are needed.

Strategies and lessons

- How to foster leadership and broad involvement. Our D&I capacity building efforts have involved a broad range of faculty, staff, and students. For WUNDIR and DIRC, the commitment of senior-level faculty members has been essential for providing technical assistance and mentoring junior scholars. The senior D&I leaders at Washington University provide significant amounts of time to building infrastructure and mentoring junior faculty, often beyond the small amounts of allocated effort. This senior-level leadership has fostered involvement and ownership of many others across all levels (from students to faculty). The close connection with junior faculty members has allowed for their career growth in areas such as methods training and mentorship [49].
- Find ways to learn and network outside of the home institution. Many budding D&I scholars will lack senior mentors and infrastructure in their own institutions. To overcome this challenge, it is helpful to become part of training programs (e.g., the Training Institute for Dissemination and Implementation Research in Health [50]), participate in ongoing webinars (e.g., the Implementation Science Webinar Series [51]), and join peer networks (e.g., the Society for Implementation Research Collaboration [52]).

### Challenge 5: the need to balance consultation and time for research

In providing significant D&I research services, it is common to devote extensive time and resources to consultation and technical support, without growing the science outside of the home institution.

Strategies and lessons

- Build the science. We have sought to focus and maintain attention on building the D&I science in ways that overlap with our service functions, nearly always involving graduate students and junior faculty members (e.g., in grants, scientific articles). These efforts have resulted in advances across numerous areas of D&I science including: models and frameworks [44, 45], D&I strategies [53–55], D&I outcomes [46], sustainability [56, 57], systems science [23, 58], dissemination planning [59–61], and the scholarship of training [5, 41, 43].

### Challenge 6: how to move beyond the walls of academe

Most of our efforts have focused on building D&I research capacity at Washington University. Many of the resulting projects involve stakeholders in low resource settings across diverse sectors. More emphasis is needed on how to build and maintain D&I research in settings with limited resources.

Strategies and lessons

- Extend the reach to stakeholders and low resource settings. Fellowship programs that link academic institutions with practice sites show promise in building D&I research capacity [62]. Building on principles of local ownership and mentoring [63], these efforts often involve training and technical assistance for practice-oriented researchers and community-level partners [64, 65]. The recently launched TRIPLE training is an example of an effort to leverage D&I research-based knowledge for training clinical leaders how to implement evidence-based practices. Washington University seeks to better bridge the clinical and research worlds by linking hospital-based quality improvement with D&I science principles.

### Challenge 7: the need to build a greater focus on evaluation

As our D&I programs have become larger and more complex, formal systems have been needed to help identify what is working well and where there are opportunities for improvement.

Strategies and lessons

- Develop data systems for evaluation. It is essential to build data bases to help monitor and evaluate D&I capacity building. For efforts such as WUNDIR, this requires data on member characteristics, meeting attendance, grants and publications, and other impacts of participation. These data can be used for process evaluation to improve functioning and to document accomplishments (ongoing quality improvement activities). The data we have collected thus far provide the foundation for a future, more comprehensive impact evaluation.

## Conclusions

Our experience in developing D&I research at Washington University in St. Louis shows how significant capacity can be built in a relatively short period of time. We believe that the approaches to capacity building outlined here needs to be an explicit objective of research institutions. As evident in this article, there are many components in our efforts—across these activities, the sum is much greater than the individual parts. While D&I capacity building is inherently difficult to evaluate [5, 10, 41, 50, 63, 66], we have shown numerous markers of success and indications that our efforts are impacting our institution and contributing to D&I science. Our initial capacity building efforts lay the foundation for a future, comprehensive impact evaluation.

Every organization is different and it is unlikely that “one size fits all” when it comes to building D&I capacity across diverse research settings and in light of local challenges. However, we believe that many of our ideas and ingredients for success can be replicated, tailored, and improved upon by others.

## Additional files

Additional file 1: 2016 WUNDIR Member Survey (PDF 86 kb)

Additional file 2: WUNDIR Evaluation: Key Informant Interview Guide (PDF 151 kb)

## Abbreviations

D&I: Dissemination and implementation
DIRC: Dissemination and Implementation Research Core
ICTS: Institute for Clinical and Translational Science
IRI: Implementation Research Institute
MT-DIRC: Mentored Training for Dissemination and Implementation Research in Cancer
QUERI: Quality Enhancement Research Initiative
TRIPLE: Training in Implementation Practice Leadership
WUNDIR: Washington University Network for Dissemination and Implementation Research
