# Supplementary material for: Building capacity for dissemination and implementation research: one university’s experience
Source: Implement Sci. 2017 Aug 16;12:104. doi: 10.1186/s13012-017-0634-4 (PMC5559847; doi:10.1186/s13012-017-0634-4)
Supplement: Supplementary file 1 — 2016 WUNDIR Member Survey (PDF 86 kb) [file 13012_2017_634_MOESM1_ESM.pdf]

Additional file 1. 2016 WUNDIR Member Survey

Select the option that best fits your job description:
- ❍ Graduate student
- ❍ Research staff
- ❍ Post-doc
- ❍ Junior faculty (e.g., Instructor, Assistant Professor)
- ❍ Senior faculty (e.g. Associate/Full Professor)
- ❍ Other: ____________________

For your current dissemination and implementation (D&I) research, what do you consider to be your primary discipline (e.g., epidemiology, mental health, public health, clinical care, social work)?

Enter the name of your unit/department (e.g., Brown School, Biology department):

[Page Break]

How many years have you been involved in WUNDIR?
- ❍ 0
- ❍ 1
- ❍ 2
- ❍ 3 +

During 2015, WUNDIR hosted 8 meetings. How many of these meetings did you attend?
- ❍ None
- ❍ 1
- ❍ 2-3
- ❍ 4 or more
- ❍ Not sure

[Page Break]

On a scale from 1 (absolute novice) to 7 (highly expert), please rate your own current level of expertise in D&I science methods and theories.

WUNDIR typically includes the following activities. How useful has each type of activity been for your D&I work in the past 12 months?

	Not at all useful	Somewhat useful	Useful	Very useful	Not applicable
Emails (e.g. resource sharing, job and funding opportunity announcements	❍	❍	❍	❍	❍
WUNDIR meetings	❍	❍	❍	❍	❍
Introductions and member news during meetings	❍	❍	❍	❍	❍
Grant reviews	❍	❍	❍	❍	❍
Publication reviews	❍	❍	❍	❍	❍
Works in progress discussions (Idea incubator)	❍	❍	❍	❍	❍
Methods and theory discussions (based on a recent article)	❍	❍	❍	❍	❍
Guest topics/lectures	❍	❍	❍	❍	❍
Informal networking and conversation	❍	❍	❍	❍	❍
Collaborations	❍	❍	❍	❍	❍
Mentoring	❍	❍	❍	❍	❍

To what extent has your participation in WUNDIR helped improve your ability to develop grants in the past 12 months?

- ❍ Greatly improved
- ❍ Improved
- ❍ Somewhat improved
- ❍ No impact
- ❍ Not applicable

To what extent has your participation in WUNDIR helped improve your publication development in the past 12 months?

- ❍ Greatly improved
- ❍ Improved
- ❍ Somewhat improved
- ❍ No impact
- ❍ Not applicable

[Page Break]

How many grants have you improved with support from WUNDIR in the past 12 months?

- ❍ None
- ❍ 1-3
- ❍ 4-6
- ❍ 7-10
- ❍ 10+

How many publications have you improved with support from WUNDIR in the past 12 months?

- ❍ None
- ❍ 1-3
- ❍ 4-6
- ❍ 7-10
- ❍ 10+

To what extent has WUNDIR helped increase your knowledge of the D&I field?

- ❍ Greatly increased
- ❍ Increased
- ❍ Somewhat increased
- ❍ Did not increase
- ❍ Not applicable

To what extent has WUNDIR helped increase your ability to network with other D&I researchers at Washington University?

- ❍ Greatly increased
- ❍ Increased
- ❍ Somewhat increased
- ❍ Did not increase
- ❍ Not applicable

[Page Break]

In the past 12 months, my involvement in WUNDIR has led to: (check all that apply)

- ❑ New proposal idea
- ❑ New paper idea
- ❑ New dissemination of D & I research
- ❑ New professional connections
- ❑ Increased knowledge on D & I field
- ❑ Increased knowledge of D & I research at WU
- ❑ New collaborations on projects
- ❑ Submission of a proposal
- ❑ Submission of a paper
- ❑ Enhanced skills in dissemination research
- ❑ Enhanced skills in implementation research
- ❑ None of the Above
- ❑ Other: ____________________

[Page Break]

In the past 12 months, what barriers have kept you from participating in WUNDIR meetings? (check all that apply)

- ❑ I don't have the time
- ❑ I don't know the meeting time, location or topics
- ❑ The meetings are too long
- ❑ The meeting locations are difficult to get to
- ❑ I'm not involved in dissemination or implementation research
- ❑ The meeting topics don’t interest me
- ❑ I have not felt comfortable at the meetings
- ❑ There has been a lack of equal participation among members
- ❑ I have not experienced any barriers to attending
- ❑ Other: ____________________

[Page Break]

What is the best part of WUNDIR that we should never change?

What is the one thing we can do to most improve WUNDIR?

In what ways can WUNDIR increase staff, student, and junior faculty involvement?

In what ways can WUNDIR strengthen D&I capacity at Washington University?
