# Supplementary material for: Building capacity for dissemination and implementation research: one university’s experience
Source: Implement Sci. 2017 Aug 16;12:104. doi: 10.1186/s13012-017-0634-4 (PMC5559847; doi:10.1186/s13012-017-0634-4)
Supplement: Supplementary file 2 — WUNDIR Evaluation: Key Informant Interview Guide (PDF 151 kb) [file 13012_2017_634_MOESM2_ESM.pdf]

Additional file 2. WUNDIR Evaluation: Key Informant Interview Guide

Introduction and Purpose

Good afternoon and thank you for taking the time to meet with me today. My name is _______ and I will be speaking with you about your experience with the Washington University Network of Dissemination and Implementation Researchers (WUNDIR). The information you provide me today will be useful to WUNDIR for expanding and improving their services. The questions I ask you will be related to your thoughts about the benefits of WUNDIR, changes for the future, and WUNDIR's impact at Washington University.

The interview will take between 20 – 30 minutes. Please do not hesitate to ask me to repeat or clarify any questions. We will be audio recording today's interview to improve note taking processes, but all of your answers will be kept confidential. Your name will not be associated with any of your comments. Lastly, there are no right or wrong answers in this interview. We value your opinions whether they are positive or negative and look forward to learning about your perspective of WUNDIR.

Do you have any questions before we begin?

Introduction

To begin, I am interested in hearing about your involvement in WUNDIR.

1. Please tell me how long you have been involved in WUNDIR and to what extent.

Benefits and Challenges

Next I want to explore some of the benefits and challenges of participating in WUNDIR.

2. *Based on our survey data, we know about some of the benefits to being involved in WUNDIR. These include increased knowledge of D&I research at WU, increased knowledge of the D&I field, and increased ability to make new professional connections.* **Are there any other benefits you have experienced that you would like to share?**

    a) *Follow-up:* What do you think has worked well with WUNDIR?

3. *Based on our survey data, we know about some of the major barriers to being involved in WUNDIR. These include not having enough time, the meeting length and not being interested in the topics presented.* **Would you think about one of those barriers that you can relate to and tell me how we might address it?**

    a) *Follow-up:* Are there any other barriers you have experienced that you would like to share?

        1. *Follow-up:* How do you suggest addressing these barriers?

b) *Follow-up:* What do you think are other ways that members could participate in WUNDIR if they cannot attend meetings?

1. *For each example given:* Would you personally take advantage of this opportunity if it were offered through WUNDIR?

    - *Probe: webinars, teleconferencing, D&I online discussion forums, shorter WUNDIR topical meetings, social gatherings*

c) *Many of the survey respondents suggested that WUNDIR could increase its marketing and outreach efforts.* **What do you think are the most effective ways to reach out to healthcare professionals, students and individuals not currently involved in WUNDIR?**

1. *Probe: improved website, flyer highlighting benefits, social media, emails, etc.*

4. **What do you think has not worked well with WUNDIR?**

<u>Improvements & New Directions</u>

Now I want to talk to you about how WUNDIR can improve its network and explore some new directions the group can take in the future.

5. **How can WUNDIR be improved?**

6. **In what ways should the WUNDIR network be expanded, strengthened, or contracted?**

<u>Building Capacity</u>

I have a few questions about your perspective on WUNDIR's impact at Washington University and within the broader field of D&I science. This information will inform how WUNDIR can advance their presence at Washington University and contribute to the D&I field.

7. **In what ways does WUNDIR strengthen D&I capacity at Washington University, if at all?**

    a) *Probe: informal impact of WUNDIR, building awareness, impact on WU curriculum*

8. **In what ways does WUNDIR build capacity for the D&I field outside of Washington University, if at all?**

    a) Probe: WU contributions to field, build national and int'l awareness, replicating the model at other universities

<u>Summary/Closing</u>

9. **Those were all of the questions I had for you today. Is there anything else you would like to share that we haven't covered?**

Thank you again for taking the time to speak with me today about WUNDIR. The Evaluation Center appreciates your input and your answers will be useful for the future direction of WUNDIR. Please don't hesitate to contact me if you have any questions related to the interview.
